# Supplementary material for: Modelling Skylarks (Alauda arvensis) to Predict Impacts of Changes in Land Management and Policy: Development and Testing of an Agent-Based Model
Source: PLoS One. 2013 Jun 6;8(6):e65803. doi: 10.1371/journal.pone.0065803 (PMC3675089; doi:10.1371/journal.pone.0065803)
Supplement: Supporting Information S4 — The skylark ODdox as a zipped archive. (ZIP) [file pone.0065803.s004.zip › Skylark_ODdox/class_skylark___base.html]

ALMaSS Skylark ODdox: Skylark\_Base Class Reference


ALMaSS Skylark ODdox
 2.0


- Main Page
- Related Pages
- Classes
- Files

- Class List
- Class Index
- Class Hierarchy
- Class Members

Public Member Functions |
Public Attributes |
Protected Member Functions

Skylark\_Base Class Reference

`#include <skylarks_all.h>`

List of all members.

|  |  |
| --- | --- |
| Public Member Functions | |
| void | AddStriglingMort (int lifestage) |
| bool | InSquare (rectangle R) |
| virtual double | On\_FoodSupply (double) |
|  | Skylark\_Base (int x, int y, SkTerritories \*Terrs, Landscape \*L, Skylark\_Population\_Manager \*SPM, int bx, int by, int mh) |
| virtual int | WhatState () |
| Public Member Functions inherited from TAnimal | |
| virtual void | BeginStep (void) |
| void | CheckManagement (void) |
| void | CheckManagementXY (int x, int y) |
| virtual void | CopyMyself () |
| virtual void | Dying () |
| virtual void | EndStep (void) |
| virtual void | KillThis () |
| virtual bool | OnFarmEvent (FarmToDo) |
| void | SetX (int a\_x) |
| void | SetY (int a\_y) |
| virtual void | Step (void) |
| int | Supply\_m\_Location\_x () |
| int | Supply\_m\_Location\_y () |
| unsigned | SupplyFarmOwnerRef () |
| int | SupplyPolygonRef () |
| AnimalPosition | SupplyPosition () |
|  | TAnimal (int x, int y, Landscape \*L) |
| Public Member Functions inherited from TALMaSSObject | |
| void | OnArrayBoundsError () |
|  | TALMaSSObject () |
| virtual | ~TALMaSSObject () |

|  |  |
| --- | --- |
| Public Attributes | |
| int | Age |
| int | m\_Born\_x |
| int | m\_Born\_y |
| TTypesOfSkState | m\_CurrentSkState |
| int | m\_MyHome |
|  | The vegetation type where the skylark was born. |
| Skylark\_Population\_Manager \* | m\_OurPopulationManager |
| SkTerritories \* | m\_OurTerritories |
| double | m\_pcide\_conc |
| double | m\_pesticide\_accumulation |
| double | m\_Size |

|  |  |
| --- | --- |
| Protected Member Functions | |
| bool | DailyMortality (int mort) |
| virtual void | PesticideResponse () |
| Protected Member Functions inherited from TAnimal | |
| void | CorrectWrapRound (void) |
|  | Corrects wrap around co-ordinate problems. |

|  |  |
| --- | --- |
| Additional Inherited Members | |
| Protected Attributes inherited from TAnimal | |
| int | m\_Location\_x |
| int | m\_Location\_y |
| Landscape \* | m\_OurLandscape |

---

## Constructor & Destructor Documentation

|  |  |  |  |
| --- | --- | --- | --- |
| Skylark\_Base::Skylark\_Base | ( | int | *x*, |
|  |  | int | *y*, |
|  |  | SkTerritories \* | *Terrs*, |
|  |  | Landscape \* | *L*, |
|  |  | Skylark\_Population\_Manager \* | *SPM*, |
|  |  | int | *bx*, |
|  |  | int | *by*, |
|  |  | int | *mh* |
|  | ) |  |  |

References TALMaSSObject::CurrentStateNo, m\_Born\_x, m\_Born\_y, m\_CurrentSkState, m\_MyHome, m\_OurPopulationManager, m\_OurTerritories, m\_pesticide\_accumulation, and toss\_Initiation.

: TAnimal( x, y, L ) {

m\_OurTerritories = Terrs;

m\_OurPopulationManager = SPM;

// Make sure that the skylark starts in the correct state

m\_CurrentSkState = toss\_Initiation;

CurrentStateNo = 0;

m\_Born\_x = bx;

m\_Born\_y = by;

m\_MyHome = mh;

m\_pesticide\_accumulation = 0;

}

---

## Member Function Documentation

|  |  |  |  |  |  |
| --- | --- | --- | --- | --- | --- |
| void Skylark\_Base::AddStriglingMort | ( | int | *lifestage* | ) |  |

inline

Referenced by Skylark\_Clutch::OnFarmEvent(), Skylark\_Nestling::OnFarmEvent(), Skylark\_PreFledgeling::OnFarmEvent(), and Skylark\_Female::OnFarmEvent().

{

m\_OurPopulationManager->AddStriglingMort( lifestage );

}

|  |  |  |  |  |  |
| --- | --- | --- | --- | --- | --- |
| bool Skylark\_Base::DailyMortality | ( | int | *mort* | ) |  |

protected

References random().

Referenced by Skylark\_Clutch::st\_Developing(), Skylark\_Nestling::st\_Developing(), and Skylark\_PreFledgeling::st\_Developing().

{

#ifdef \_\_CJTDebug\_5

if ( IsAlive() != 0x0DEADC0DE ) DEADCODEError();

#endif

// Timing: Must be called once each day and if true then death

//returns true if the object must die

int ch = random( 10000 );

if ( ch < mort ) return true; else

return false;

}

|  |  |  |  |  |  |
| --- | --- | --- | --- | --- | --- |
| bool Skylark\_Base::InSquare | ( | rectangle | *R* | ) |  |

References TAnimal::m\_Location\_x, TAnimal::m\_Location\_y, rectangle::m\_x1, rectangle::m\_x2, rectangle::m\_y1, and rectangle::m\_y2.

{

#ifdef \_\_CJTDebug\_5

if ( IsAlive() != 0x0DEADC0DE ) DEADCODEError();

#endif

if ( ( m\_Location\_x >= int( p\_R.m\_x1 ) ) && ( m\_Location\_x < int( p\_R.m\_x2 ) ) && ( m\_Location\_y >= int( p\_R.m\_y1 ) )

&& ( m\_Location\_y < int( p\_R.m\_y2 ) ) )

return true; else

return false;

}

|  |  |  |  |  |  |
| --- | --- | --- | --- | --- | --- |
| virtual double Skylark\_Base::On\_FoodSupply | ( | double |  | ) |  |

inlinevirtual

Reimplemented in Skylark\_Nestling.

{

return 0.0;

}

|  |  |  |  |  |  |
| --- | --- | --- | --- | --- | --- |
| virtual void Skylark\_Base::PesticideResponse | ( | void |  | ) |  |

inlineprotectedvirtual

Reimplemented in Skylark\_Male, Skylark\_Female, Skylark\_PreFledgeling, and Skylark\_Nestling.

{ return; }

|  |  |  |  |  |
| --- | --- | --- | --- | --- |
| virtual int Skylark\_Base::WhatState | ( |  | ) |  |

inlinevirtual

Reimplemented from TAnimal.

Referenced by Skylark\_Population\_Manager::ProbePOM().

{

return m\_CurrentSkState;

}

---

## Member Data Documentation

int Skylark\_Base::Age

Referenced by Skylark\_Male::DefendTerritory(), Skylark\_PreFledgeling::GetFood(), Skylark\_Male::GetMigrationMortality(), Skylark\_Nestling::On\_FoodSupply(), Skylark\_PreFledgeling::OnFarmEvent(), Skylark\_Adult::Skylark\_Adult(), Skylark\_Clutch::Skylark\_Clutch(), Skylark\_Nestling::Skylark\_Nestling(), Skylark\_PreFledgeling::Skylark\_PreFledgeling(), Skylark\_Male::st\_CaringForYoung(), Skylark\_Clutch::st\_Developing(), Skylark\_Nestling::st\_Developing(), Skylark\_PreFledgeling::st\_Developing(), Skylark\_Female::st\_Emigrating(), Skylark\_Male::st\_Emigrating(), Skylark\_Male::st\_FindingTerritory(), Skylark\_Female::st\_Immigrating(), Skylark\_Male::st\_Immigrating(), Skylark\_Nestling::st\_Maturing(), Skylark\_Male::Step(), and Skylark\_Population\_Manager::TheFledgelingProbe().

int Skylark\_Base::m\_Born\_x

Referenced by Skylark\_Base(), Skylark\_Male::st\_Arriving(), Skylark\_Clutch::st\_Hatching(), Skylark\_Nestling::st\_Maturing(), and Skylark\_PreFledgeling::st\_Maturing().

int Skylark\_Base::m\_Born\_y

Referenced by Skylark\_Base(), Skylark\_Male::st\_Arriving(), Skylark\_Clutch::st\_Hatching(), Skylark\_Nestling::st\_Maturing(), and Skylark\_PreFledgeling::st\_Maturing().

TTypesOfSkState Skylark\_Base::m\_CurrentSkState

Referenced by Skylark\_Female::BeginStep(), Skylark\_Male::BeginStep(), Skylark\_Population\_Manager::BreedingPairsOutput(), Skylark\_Clutch::EndStep(), Skylark\_Nestling::EndStep(), Skylark\_PreFledgeling::EndStep(), Skylark\_Male::EndStep(), Skylark\_Female::OnBroodDeath(), Skylark\_Male::OnBroodDeath(), Skylark\_Female::OnClutchDeath(), Skylark\_Nestling::OnDeserted(), Skylark\_Male::OnEggHatch(), Skylark\_Female::OnEggsHatch(), Skylark\_Male::OnEvicted(), Skylark\_Clutch::OnFarmEvent(), Skylark\_Nestling::OnFarmEvent(), Skylark\_PreFledgeling::OnFarmEvent(), Skylark\_Female::OnFarmEvent(), Skylark\_Male::OnFarmEvent(), Skylark\_Female::OnMateDying(), Skylark\_Male::OnMateDying(), Skylark\_Female::OnMateHomeless(), Skylark\_Male::OnMateLeaving(), Skylark\_Clutch::OnMumGone(), Skylark\_Male::OnPairing(), Skylark\_Male::OnPreFledgelingMature(), Skylark\_Male::OnReHouse(), Skylark\_Female::OnStopFeedingChicks(), Skylark\_Nestling::OnYouHaveBeenEaten(), Skylark\_Base(), Skylark\_PreFledgeling::Skylark\_PreFledgeling(), Skylark\_Clutch::st\_Dying(), Skylark\_Nestling::st\_Dying(), Skylark\_PreFledgeling::st\_Dying(), Skylark\_Female::st\_Dying(), Skylark\_Male::st\_Dying(), Skylark\_Male::st\_FollowingMate(), Skylark\_Clutch::Step(), Skylark\_Nestling::Step(), Skylark\_PreFledgeling::Step(), Skylark\_Female::Step(), and Skylark\_Male::Step().

int Skylark\_Base::m\_MyHome

The vegetation type where the skylark was born.

Referenced by Skylark\_Base(), Skylark\_Clutch::st\_Hatching(), Skylark\_Nestling::st\_Maturing(), Skylark\_PreFledgeling::st\_Maturing(), and Skylark\_Population\_Manager::TheFledgelingProbe().

Skylark\_Population\_Manager\* Skylark\_Base::m\_OurPopulationManager

Referenced by Skylark\_Male::EstablishingATerritory(), Skylark\_Female::EstablishTerritory(), Skylark\_PreFledgeling::GetFledgelingEM(), Skylark\_PreFledgeling::GetFood(), Skylark\_Male::GetMigrationMortality(), Skylark\_Nestling::OnDeserted(), Skylark\_Male::OnEvicted(), Skylark\_Female::OnMateDying(), Skylark\_Female::OnMateHomeless(), Skylark\_Nestling::OnYouHaveBeenEaten(), Skylark\_Adult::RemoveEM(), Skylark\_Base(), Skylark\_Male::st\_Arriving(), Skylark\_Clutch::st\_Developing(), Skylark\_Nestling::st\_Developing(), Skylark\_Female::st\_Dying(), Skylark\_Female::st\_Floating(), Skylark\_Male::st\_Floating(), Skylark\_Female::st\_GivingUpTerritory(), Skylark\_Clutch::st\_Hatching(), Skylark\_Female::st\_Incubating(), Skylark\_Female::st\_Laying(), Skylark\_Nestling::st\_Maturing(), Skylark\_PreFledgeling::st\_Maturing(), Skylark\_Female::st\_StartingNewBrood(), Skylark\_Female::st\_StoppingBreeding(), Skylark\_Female::Step(), and Skylark\_Male::Step().

SkTerritories\* Skylark\_Base::m\_OurTerritories

Referenced by Skylark\_Male::ConstructAHabitatTable(), Skylark\_Male::EstablishingATerritory(), Skylark\_Female::EstablishTerritory(), Skylark\_Male::OnEvicted(), Skylark\_Female::OnMateDying(), Skylark\_Male::OnMateLeaving(), Skylark\_Male::OnReHouse(), Skylark\_Male::ReEvaluateTerritory(), Skylark\_Base(), Skylark\_Male::st\_AttractingAMate(), Skylark\_Female::st\_Dying(), Skylark\_Male::st\_Dying(), Skylark\_Female::st\_Finding\_Territory(), Skylark\_Male::st\_FindingTerritory(), Skylark\_Male::st\_FollowingMate(), Skylark\_Female::st\_GivingUpTerritory(), Skylark\_Clutch::st\_Hatching(), Skylark\_Female::st\_Laying(), Skylark\_Nestling::st\_Maturing(), Skylark\_PreFledgeling::st\_Maturing(), and Skylark\_Female::st\_StoppingBreeding().

double Skylark\_Base::m\_pcide\_conc

Referenced by Skylark\_Female::FeedYoung(), Skylark\_Adult::RemoveEM(), and Skylark\_Male::st\_CaringForYoung().

double Skylark\_Base::m\_pesticide\_accumulation

Referenced by Skylark\_Adult::RemoveEM(), Skylark\_Base(), and Skylark\_Male::st\_CaringForYoung().

double Skylark\_Base::m\_Size

Referenced by Skylark\_Nestling::On\_FoodSupply(), Skylark\_Adult::Skylark\_Adult(), Skylark\_Nestling::Skylark\_Nestling(), Skylark\_PreFledgeling::Skylark\_PreFledgeling(), Skylark\_Male::st\_CaringForYoung(), Skylark\_Nestling::st\_Developing(), Skylark\_PreFledgeling::st\_Developing(), Skylark\_Nestling::st\_Maturing(), and Skylark\_PreFledgeling::st\_Maturing().

---

The documentation for this class was generated from the following files:

- skylarks\_all.h
- skylarks\_all.cpp


- Skylark\_Base
- Generated on Thu Jan 10 2013 13:15:37 for ALMaSS Skylark ODdox by
   1.8.1.1
